# Case Report: Orbital metastasis as the presenting feature of lung cancer

**DOI:** 10.12688/f1000research.11247.1

**Published:** 2017-04-05

**Authors:** Sunil Munakomi, Samrita Chaudhary, Pramod Chaudhary, Jagdish Thingujam, Bijoy Mohan Kumar, Iype Cherian

**Affiliations:** 1 Department of Neurosurgery, Nobel Teaching Hospital, Biratnagar, Nepal

**Keywords:** orbit, metastasis, lung cancer

## Abstract

Orbital metastasis from lung cancer as an initial presenting symptom is a rare entity, which may paradoxically delay the diagnosis and initiation of correct management, due to the confusion of it being primary orbital pathology. Herein we report a case of a 58 year old woman, who presented with painful orbital swelling along with diminution in her vision. The patient was initially thought to have a primary eye lesion; however chest X-ray was suggestive of a lung mass, which was confirmed by chest computed topography followed by ultrasound guided fine needle aspiration cytology. The patient was then referred to a cancer centre for further management. This case report aims to increase the knowledge about this metastasis as a probable cause of orbital symptoms in certain subsets of patients, so that correct therapeutic decisions may be made in the future.

## Introduction

Orbital metastatis as the initial presenting symptom from a metastatic lung lesion is a rare entity, occurring at an incidence of approximately 7% ^1, 2^. However, this should be kept as one of the differentials in any patients presenting with orbital symptoms, so as to frame an accurate and effective plan of management. Occasionally such rare presentations would invariably lead to a delay in the correct diagnosis, thereby increasing the risk of loss of vision, which decreases the quality of life of patients ^3^. Poor management also increases the odds of progressing the tumor stage. Herein, we report one such case in a 58 year old woman, who presented with unilateral peri-orbital swelling and diminution of vision. Following detailed examination and investigations, the patient was found to harbor a malignant lung lesion.

## Case report

**Figure 1.**
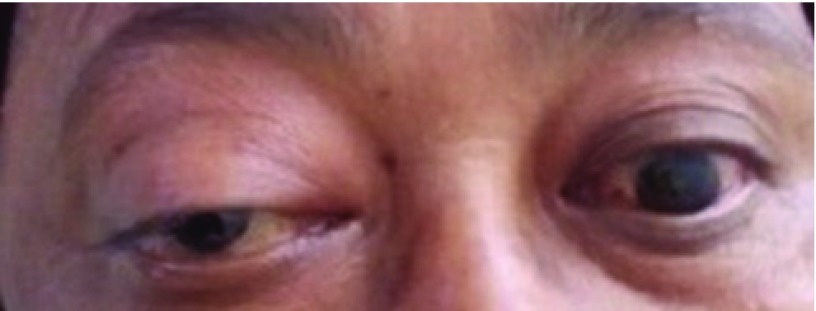
Image showing right sided peri-orbital swelling.

A 58 year old woman from central Nepal presented to our outpatient clinic with a history of painful swelling around her right eye for two months. The patient also complained of diminishing vision in the same eye. The vision in the patient’s left eye had been previously lost following an injury during childhood. There was no other relevant family information or any significant past medical or surgical illnesses of the patient. Local examination revealed presence of peri-orbital swelling in the right eye with restricted eye movements ( Figure 1). The patient’s visual acuity in the same eye was restricted to only perception to light. Funduscopy revealed the presence of papilledema. Remaining physical examinations were normal.

**Figure 2.**
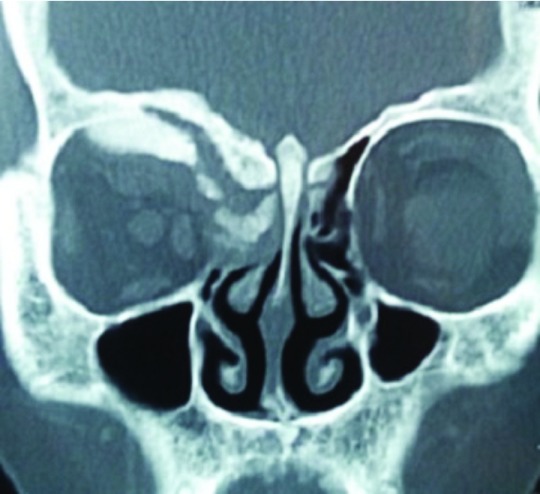
Image showing abnormal hyperostotic changes within the right orbit in the computed tomography scan.

**Figure 3.**
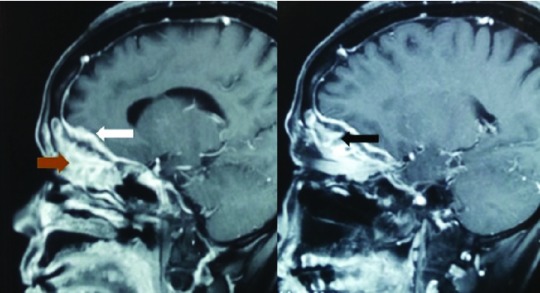
Image showing enhancing lesion in the right globe (black arrow) with extension to para-nasal sinuses (brown arrow) and invasion to the dura of anterio cranial fossa (white arrow).

Radio-imaging of the patient’s orbits revealed the presence of hyperostotic changes in theright orbit, with presence of enhancing lesions on the right globe with extension to the para-nasal sinuses and also invasion along the dural base in the anterior cranial fossa ( Figure 2 and Figure 3). The initial differential diagnosis was an infective pathology. However, the patient was not immuno-compromised.

**Figure 4.**
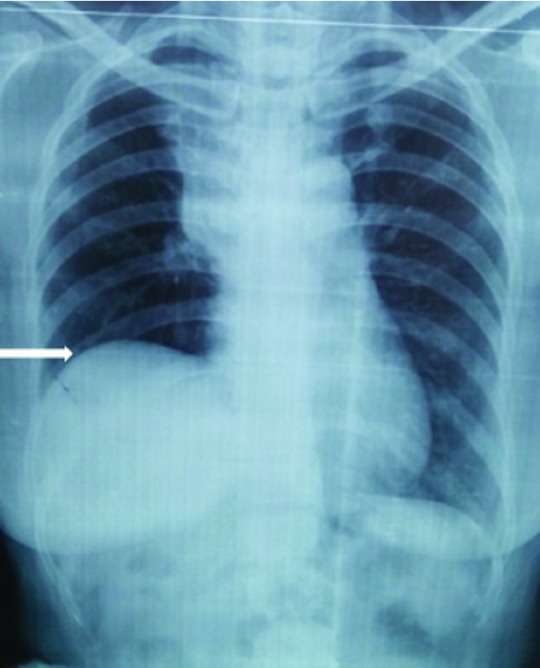
Chest X-ray showing elevated right hemi-diaphragm (white arrow) and right para-hilar mass.

**Figure 5.**
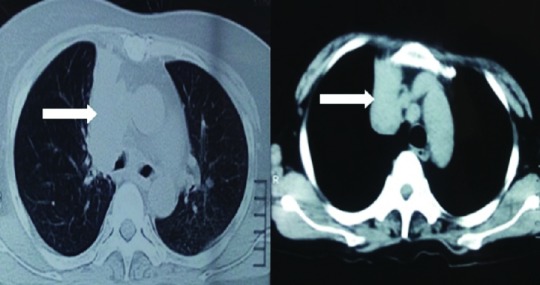
Chest computed tomography revealing presence of right para-hilar lung mass (white arrows).

A chest X-ray was performed as a routine work up, which inadvertently revealed the presence of an elevated right hemi-diaphragm with presence of right para-hilar mass ( Figure 4). Further evaluation through chest computed tomography confirmed the finding of a right para-hilar mass ( Figure 5).

**Figure 6.**
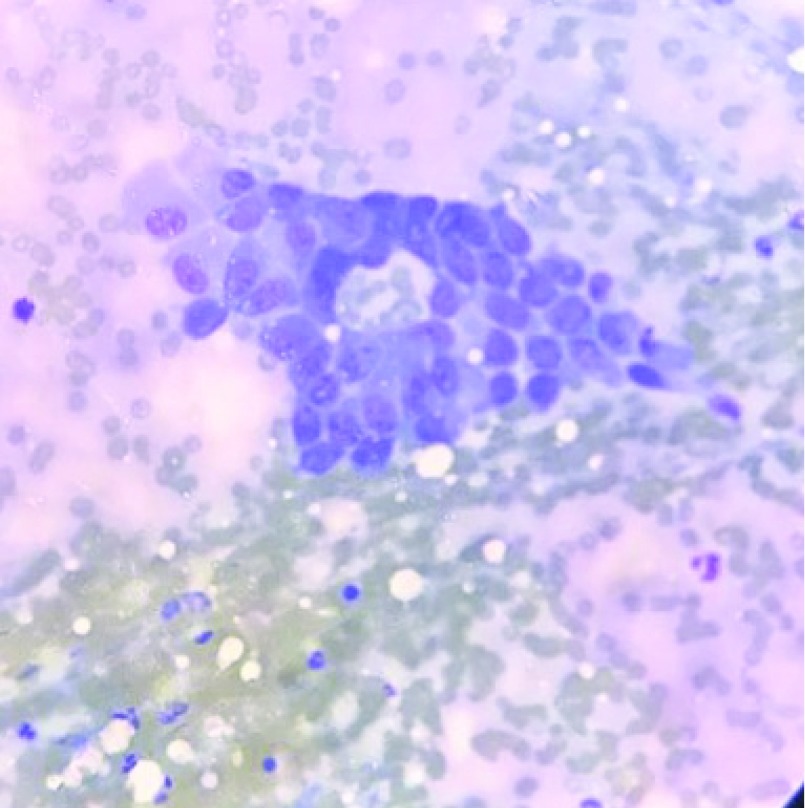
Fine needle aspiration cytology from the lung mass revealing the presence of malignant cells.

**Figure 7.**
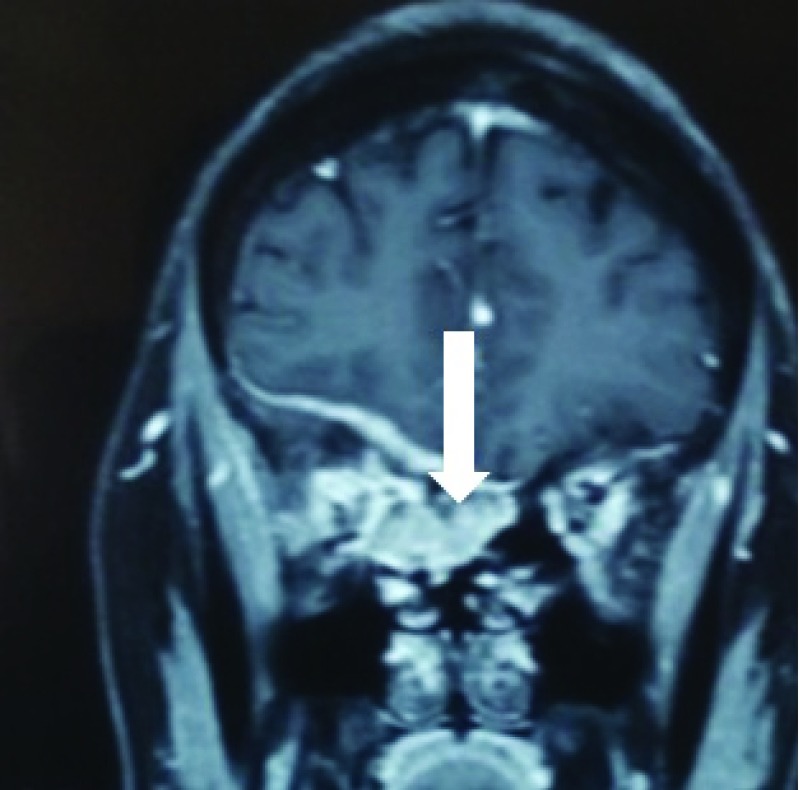
Computed tomography image revealing the presence of invasion of para-nasal sinuses by the lesion.

We discussed with the patient and her relatives the possibility of the eye findings to be related to the lung lesion and recommended approaches to obtain a definitive diagnosis. Ultrasound guided fine needle aspiration cytology (FNAC) from the lung lesion revealed findings suggestive of a malignant lung disease ( Figure 6). Diagnostic biopsy from the nasal endoscope confirmed the metastatic nature of the disease from the lung ( Figure 7). Therefore, a diagnosis of metastatic lung disease to the orbit was finally confirmed.

The patient was started on a steroid therapy (injection dexamethasone at 8mg stat followed by 4 mg every eight hours), which decreased the swelling on the patient’s eye and improved visual acuity to finger counting within a period of 1 week. This further hinted at compressive rather than infiltrative effect on the optic nerve by the lesion. The patient was counseled and then immediately referred to the National Cancer Centre, Kathmandu, Nepal for further management with systemic chemo-radiation therapy after evaluation. Since the patient had a single and minimally functioning eye left, the decision was taken not to surgically decompress the lesion from the orbit. The patients was initially started on chemotherapy with a further plan of management to be tailored as per the clinical response seen in the patient.

Initially, metastatic deposits causing eye swelling in the patient was not suspected. It was serendipity that the routine chest X-ray gave a clue to the presence of a lung mass. Even a small delay may have had a disastrous impact on the outcome of the vision in the patient.

## Discussion

Metastatic disease to the orbit is a rare epiphenomenon occurring in only 7% of all cancers ^1– 2^. Of these, symptoms related to orbital metastasis presents earlier to that of the primary lesion in around 20% of patients ^2^. Breast, prostate and lung carcinomas are the usual primaries in many cases of metastatic lesions to the orbit ^4– 5^. Lid swelling are a common presentation in such metastatic lesions ^5^, which can paradoxically delay the actual diagnosisaccounting for the benign orbital lesions. Diplopia is the most common presenting symptom in metastatic lesions, while proptosis or visual loss is seen in patients with primary orbital neoplasms ^6^. Loss of vision can be due to either direct infiltration to the optic nerve or subsequent to the mass effect. Rarely, is it subsequent to paraneoplastic phenomenon mainly from lung carcinoma. Pain resulting from perineural invasion is typical for metastatic orbital lesions ^6^.

Diagnosis can be confirmed with FNAB, which has a diagnostic accuracy of more than 90% ^7^. Further investigations need to be carried out to stage the tumor before embarking on the management option; PET scan is a rapid viable model for assessment tumor staging ^8^.

Surgical debulking is the cornerstone of management in patients with diminished vision subsequent to optic nerve compression. This was not attempted in our case, since it was the only functioning eye in the patient and that was functionally impaired as well. Surgical removal of the lesion may be locally effective in few patients having symptoms, due to compression on the optic nerve following raised intra –orbital pressure ^6^. However, chemo-radiation is usually preferred to surgery because it is non-invasive. Chemotherapy, especially platinum base regimes, is chosen for small cell lung cancer over radiation because of the risk of damage to the eye lens. For non-small cell cancers, either photon radiation of 30–40 Gy, or newer frontiers, such as tyrosine kinase inhibitors, are the mainstay of treatment. However, overall prognosis, despite systemic therapy, is poor with a median survival of little over 1 year, and only 27% of patients surviving for more than two years ^6, 9– 13^. Compared to breast cancer, lung cancers metastasize early to the orbit and also have shorter median survival time ^14^.

## Conclusions

It is prudent to provide a strategy for management of cases presenting with eye symptoms, so that rare causes, such as metastatic lesions, are not omitted. Such a strategy would certainly help in providing an early and effective treatment plan in such patients with metastatic orbital lesions. This would increase the chance of improving vision, escalate quality of life and also initiate early cancer therapy following appropriate work up and staging.

## Consent

Both written and verbal informed consent for publication of images and clinical data related to this case was sought and obtained from the patient.
