# Supplementary material for: Case Report: Orbital metastasis as the presenting feature of lung cancer
Source: F1000Res. 2017 Apr 5;6:430. [Version 1] doi: 10.12688/f1000research.11247.1 (PMC5437950; doi:10.12688/f1000research.11247.1)
Supplement: Peer review history

# Peer review: Referee response for version 1

**DOI:** 10.5256/f1000research.12134.r22711

**Published:** 2017-05-17

**Authors:** Luis Rafael Moscote-Salazar, Guru Dutta Satyarthee

**Affiliations:** 1 Department of Neurosurgery, RED LATINO (Latin American Trauma & Intensive Neurocare Organization), Bogotá, Colombia; 2 Department of Neurosurgery, Neurosciences Centre, All India Institute of Medical Sciences (AIIMS), New Delhi, Delhi, India

An interesting case of orbital metastasis of lung adenocarcinoma is presented. Lung cancer is one of the leading causes of death globally. Adenocarcinoma occupies the first place in epidemiological frequency (50%) is also one of the most frequent types of tumors in non-smokers. They are classified into 4 histological types: acinar, papillary, bronchialveolar and the mucin secretory variety.

 The adenocarcinoma originates from mucoproductive cells. Adenocarcinoma metastasis to the orbit is infrequent. In the orbits most of the tumors are primary, but they can also reach the orbit by contiguity. At least 50% of patients with orbital metastasis are unaware of the existence of a primary tumor. Metastasis is less frequent than ocular metastasis. Apparently, there is no predilection for any specific orbit and their bilateral appearance is rare. The most frequent orbital metastasis are breast, lung and orbit. They have been described a 5 types of clinical syndromes associated with orbital metastasis. The first type is mass syndromes.

 In more than 50% of cases. This syndrome causes displacement of the eyeball. The second type is infiltrative. The third type is an inflammatory type. The fourth type of metastasis is called functional and is frequently located in apex orbital. The last type is silent, does not produce symptomatology. The use of fine needle aspiration biopsy is an excellent option when orbital metastasis is suspected. Finally, the patients with orbital metastasis are not candidates for orbit surgery for extirpation of the tumor mass. The realization of surgery does not offer a cure. In cases of slow-growing tumors, the extirpation of metastasis and the primary tumor may improve the prognosis. Management strategies include radiation therapy. Sometimes the use of radiation therapy results in vision recovery.

We have read this submission. We believe that we have an appropriate level of expertise to confirm that it is of an acceptable scientific standard.

# Peer review: Referee response for version 1

**DOI:** 10.5256/f1000research.12134.r22208

**Published:** 2017-05-04

**Authors:** Trevor K. Rogers

**Affiliations:** 1 Chest Clinic, Doncaster Royal Infirmary, Doncaster, UK

A major deficiency of the report is the lack of characterization of the tumour. Whilst the CT imaging is convincing for a lung primary it should at the very least be commented whether the tumour was non-small cell (and ideally whether adeno-, squamous or neuroendocrine) or small cell -the H&E slide is suspicous of the latter, although I am not a pathologist. Ideally it would have been established if it were TTF1 positive supporting a lung origin.

 I am surprised that the smoking status of the patient is not reported.

 The sentence: "Orbital metastatis as the initial presenting symptom from a metastatic lung lesion is a rare entity, occurring at an incidence of approximately 7%" - the denominator 7% of what? - I suspect orbital tumours. Metastasis spelt incorrectly

 The sentence: "Poor management also increases the odds of progressing the tumor stage." is incorrect: the disease stage is already IV. What I think they mean is that patients may become too unwell for anticancer treatments and the visual impairment worse and harder to palliate.

 Funduscopy should be fundoscopy

 "PET scan is a rapid viable model for assessment tumor staging8" PET scanning would only be considered if simpler imaging modalities failed to identify a primary site, as in a lung cancer, staging is already M1b

 I remain troubled by the 7% figure which is repeated in the discussion. If orbital metastasis really occurred in 7% of all cancers it would not be regarded as that rare. Reference 2 does not give any evidence for the 7% figure quoted, does not give the denominator (either) and I can't see any evidence for the 7% figure quoted in reference 1.

 The outcome for the patient is not described with sufficient detail, including length of survival.

I have read this submission. I believe that I have an appropriate level of expertise to confirm that it is of an acceptable scientific standard, however I have significant reservations, as outlined above.

# Peer review: Referee response for version 1

**DOI:** 10.5256/f1000research.12134.r22210

**Published:** 2017-05-02

**Authors:** Matteo Giaj Levra

**Affiliations:** 1 Thoracic Oncology Unit, Centre Hospitalier Universitaire de Grenoble, Grenoble, France

This is an interesting case report, about an unusual site of metastasis in lung cancer patient. There are details about the symptoms, diagnosis and symptomatic treatment of the orbital metastasis. 

 I would only add if there was a history of tobacco exposure (not cited in the text).

I have read this submission. I believe that I have an appropriate level of expertise to confirm that it is of an acceptable scientific standard.
